# Health literacy of students in Germany – Results of the HBSC study 2022

**DOI:** 10.25646/11870

**Published:** 2024-03-04

**Authors:** Saskia Sendatzki, Ronja Maren Helmchen, Irene Moor, Gorden Sudeck, Kevin Dadaczynski, Katharina Rathmann, for the HBSC Study Group Germany

**Affiliations:** 1 Fulda University of Applied Sciences, Department of Health Sciences; 2 Fulda University of Applied Sciences, Fulda Public Health Centre; 3 Martin Luther University Halle-Wittenberg, Halle (Saale), Medical Faculty, Interdisciplinary Centre for Health Sciences, (PZG), Institute of Medical Sociology; 4 Eberhard Karls University of Tübingen, Institute of Sports Science; 5 Eberhard Karls University of Tübingen, Interfaculty Research Institute for Sport and Physical Activity; 6 Leuphana University Lüneburg Center for Applied Health Sciences

**Keywords:** CHILDREN, ADOLESCENTS, SCHOOLS, HEALTH LITERACY, PSYCHOSOMATIC COMPLAINTS, SOCIAL STATUS, PREVALENCES, HBSC, SURVEY, GERMANY

## Abstract

**Background:**

Health literacy (HL) encompasses knowledge and skills for dealing critically and confidently with health information in individual and social contexts. Current studies show that a high proportion of children and adolescents have limited health literacy, depending on aspects of their social background. Health literacy is considered an important factor influencing health. Little is known about the development of health literacy over time and its connection with psychosomatic complaints in young people.

**Methods:**

Based on the results of the Health Behaviour in School-aged Children (HBSC) study, this article focusses on the level of HL in 11-, 13-, and 15-year-old students (N = 6,475) over time and taking social differences into account. Finally, the relationship between HL and psychosomatic complaints is analysed. Univariate, bivariate, and multivariate analyses were carried out for this purpose.

**Results:**

At 24.4 %, slightly more students have low HL in 2022 than in 2017/18 (21.4 %). There are differences in HL according to gender, age, type of school, and family affluence. Low HL is associated with a high psychosomatic burden.

**Conclusions:**

The results highlight the need for target group-specific measures to promote young people’s HL, which address individual and organisational aspects of HL.

## 1. Introduction

In recent years, an increasing amount of research has focussed on the concept of health literacy (HL). Paakkari and Paakkari [1] define health literacy as a set of five core competences in relation to dealing with health information. These include theoretical and practical knowledge, critical thinking, self-awareness, and citizenship. Citizenship describes, among other things, the ability to look at health issues beyond one’s own perspective in terms of social responsibility [1]. With regard to the COVID-19 pandemic, observing hygiene measures, wearing masks, and maintaining social distancing are some examples of citizenship.

For the target group of children and adolescents in Germany, initial studies show that between 15 and 50 per cent of adolescents have difficulties in dealing with health information [2–6]. Major challenges arise in the search for health information, especially digitally [7, 8]. Even more pronounced are difficulties in the critical evaluation and application of the health information found [3, 5, 8]. With an increasing amount and heterogeneity of information available (infodemic [9]), it is necessary to analyse regularly how HL is developing in the general population. Especially for children and adolescents, research on time trends in HL is still in its infancy. The results of the Health Literacy Survey Germany 2 (HLS-GER 2) show that the general population’s HL has had a downward tendency between 2014 and 2021. The increasing complexity of the healthcare system, digitalisation, and the rapid spread of information of varying quality are possible reasons that could have contributed to this development. Most recently in 2020, there was a slight decline in the proportion of people with a low level of HL, which could be related to the COVID-19 pandemic. People have been sensitised to the topic of health and specifically to infection protection and are increasingly engaging with health information as a result [10].

Based on the available studies, there are clear indications of an unequal distribution of HL in children and adolescents. In terms of age- and gender-specific differences, the evidence is heterogeneous, with the findings of the Health Behaviour in School-aged Children (HBSC) study 2017/18 indicating lower HL in boys and younger students [6, 11]. Furthermore, a history of migration is associated with limitations in HL [12]. Studies also point to socioeconomic inequalities in HL following a social gradient. For example, grammar school students and those with a high level of family affluence also have higher HL [2, 6, 11, 12].

Furthermore, previous studies have established correlations between HL and various health indicators [3, 4, 11]. It should be noted that, in addition to HL, aspects of the social and educational background are also associated with both health and HL indicators [13]. Even after adjustment for subjective social status and gender, adolescents with limited HL are more likely to exhibit poor dietary and physical activity behaviour, although the corresponding correlations prove to be differential, i.e. in some cases only appear for individual dimensions of HL [3, 11]. Little research has been conducted to date on the relationships between HL and the mental and psychosomatic health of children and adolescents. Zhang et al. [14] show that, in a Chinese study, adolescents with low HL exhibit more physical and psychological symptoms compared to respondents with high HL. As far as the authors are aware, no evidence is currently available for Germany on the relationship between HL and the occurrence of psychosomatic complaints in childhood and adolescence. For Germany, data from the HBSC study 2017/18 showed moderate correlations between HL and mental health problems [11].

Psychosomatic complaints are characterised by physical symptoms (both with and without organic or functional findings), which are often caused by psychological factors [15]. The latest HBSC survey 2017/18 showed that 26.9 % of children and adolescents report multiple psychosomatic complaints, i.e. symptoms occurring at least twice a week (headaches, stomach ache, backache, feeling low, irritability, nervousness, sleeping difficulties, and dizziness) [16]. With regard to gender- and age-specific differences, it is clear that girls and older children and adolescents more frequently report psychosomatic complaints as well as limitations to their mental health [17, 18]. More details on this can be found in the article by Reiß & Behn et al. [19] in this issue of the Journal of Health Monitoring.

Against this background, this article examines three research questions:

1. How does the HL of children and young people in 2022 compare to the 2017/18 school year?
2. What sociodemographic and socioeconomic differences are evident in the HL of children and young people?
3. What associations exist between HL and the psychosomatic complaints in children and adolescents?

**HBSC 2022**

**Data holder:** HBSC Study Group Germany

**Objective:** The aim of the study is to analyse the health and health behaviour of students. Continuous health monitoring through the HBSC study contributes to informing decision-makers in policy and practice about the current fields in prevention and health promotion in childhood and adolescence. A particular focus is on the influencing factors and the social contexts of health in the young generation.

**Study design:** Cross-sectional survey by written questionnaire every four years

**Population:** Students with average ages 11, 13, and 15

**Sampling:** Observation units are schools and the class groups clustered within them. From the population of all state general education schools in Germany, a cluster sample was drawn. In order to obtain a representative estimate (close to the distribution of the population), school size and the percentage distribution of students were included in the sampling, stratified by school type and federal state (Probability Proportional to Size (PPS) design).

**Data collection period:** March – November 2022

**Sample size:**

**2022:** 6,475 students

**All four survey cycles (2009/10 – 2022):** 21,788 students

**HBSC survey cycles:**

**Included in the articles in this issue of the Journal of Health Monitoring:**

▶ 2009/10
▶ 2013/14
▶ 2017/18
▶ 2022

More information can be found at https://hbsc-germany.de/ (German)

## 2. Methods

### 2.1 Sample design and study implementation

The Health Behaviour in School-aged Children (HBSC) study is designed as a cross-sectional study that takes place every four years in a school setting and surveys students aged around 11, 13, and 15 (mean deviation of 0.5 years). In Germany, these age groups mainly comprise grades 5, 7, and 9. Students at general education schools in all 16 federal states in Germany have been surveyed in the school years 2009/10, 2013/14, 2017/18, and in the calendar year 2022 as part of the HBSC study. The schools approached for participation were drawn as a cluster sample from the population of all state general education schools in Germany. In order to obtain a representative estimate (close to the distribution of the population), school size and the percentage distribution of students were included in the sampling, stratified by school type (Probability Proportional to Size (PPS) design).

The HBSC study is conducted by means of a questionnaire, which the students complete themselves. The study has been approved by the responsible ministries or state education authorities in all federal states (except North Rhine-Westphalia, as the decision of participation lies within the schools in this federal state).

Two survey cycles of the HBSC study Germany were analysed for this evaluation: the surveys in the 2017/18 school year (n = 4,347) and in 2022 (n = 6,475). In contrast to the other publications in this issue, only data from the last two survey cycles could be included in this article, as the HL has only been included in the questionnaire since the 2017/18 school year.

All data sets were standardised and adjusted by the international HBSC consortium so that the age groups are comparable. Further information on the HBSC study and the methodology can be found in the article by Winter & Moor et al. [20] in this issue of the Journal of Health Monitoring.

### 2.2 Survey and evaluation procedures

This article focuses on HL. It is considered in connection with sociodemographic and socioeconomic characteristics (gender, age, history of migration, type of school, family affluence) and psychosomatic complaints.

The Health Literacy for School-Aged Children (HLSAC) scale, which has been validated as a survey instrument for the age groups in question and is based on the understanding of HL described above [1, 11, 21, 22], was used for the students’ self-assessment of HL. The HLSAC scale comprises ten items, which were answered on a four-point Likert scale from ‘not at all true’ to ‘absolutely true’. Five components of HL (theoretical knowledge, practical knowledge or skills, critical thinking, self-awareness, and citizenship) were mapped with two items each, for which a statistical test using factor analysis confirmed that the pairs of items each belonged to one dimension of HL All items were introduced with ‘I am sure that…’, followed by various assessments (e.g. ‘…I can compare health information obtained from different sources’). To form the scale, a sum score of all items was formed, which can assume a value range between 10 and 40. This was only done for cases with complete answers to all items. The reliability of the scale was at a high level for the present sample (Cronbach’s α = 0.887). In the course of the analyses, the sum score was divided into three categories, which represent low (10 – 25), moderate (26 – 35), and high (36 – 40) HL of the respon dents. This procedure was chosen based on the analysis in other publications and to ensure the international comparability of the results [6]. When using HL as the dependent variable in the binary-logistic regression analysis (see 2.3 Statistical methods), a dichotomisation into the characteristics ‘low’ and ‘moderate/high’ was carried out, based on the original categorisation according to Paakkari et al. [6].

The HBSC Symptom Checklist (HBSC-SCL) [23] was employed to record psychosomatic complaints. It uses a five-point response scale (‘about every day’ to ‘rarely or never’) to determine how often students had experienced headaches, stomach ache, backache, feeling low, irritability or bad temper, nervousness, sleeping difficulties, and dizziness in the last six months. The answers were summarised into an index, which was then divided into two categories: according to this, a high level of psychosomatic complaints was present if students reported at least two weekly complaints in the last six months. A low level was assumed for respondents with less than two weekly complaints in the last six months. This categorisation was chosen in line with previous research and to ensure the consistency of the results. Further information on the HBSC-SCL can be found in the article by Reiß & Behn et al. [19].

Gender was recorded in the 2022 survey year using the three options ‘girl’, ‘boy’, or ‘diverse’. In the previous survey cycles, gender was recorded in binary form (girl, boy). The age was determined at the time of the survey using the information provided by the students on their month and year of birth and summarised with a deviation of +/- 0.5 years into the age categories ‘11 years’, ‘13 years’, and ‘15 years’. The students’ history of migration was operationalised via their own country of birth and the country of birth of their mother and father. Children and adolescents who had one parent not born in Germany were categorised as having a one-sided history of migration. A two-sided history of migration was recorded if either the students themselves and at least one parent or both parents were not born in Germany [20, 24].

The school types were recorded by the schools when they confirmed their participation in the study. This resulted in the following categorisation: 1. grammar school, 2. 5th and 6th grade middle school, 3. secondary general school, 4. intermediate school, 5. comprehensive school, 6. combined secondary general and intermediate school.

Family affluence was measured using the Family Affluence Scale (FAS) [25, 26]. The students were asked about material wealth indicators of their parents’ home (presence of computer, car, own room, bathroom, dishwasher, as well as vacations taken). An index was formed from these six items, which was transformed using a RIDIT (Relative to an Identified Distribution Integral Transformation) calculation and then categorised into three groups along quintiles. These categories are divided into low (< 20 %), medium (20–80 %), and high (> 80 %) family affluence.

### 2.3 Statistical methods

The evaluation included univariate, bivariate, and multivariate analyses using IBM SPSS Statistics software (version 28). In the univariate analyses, absolute and relative frequencies were used to describe the sample and the level of HL and psychosomatic complaints. A weighting factor was created for all survey cycles to ensure nationwide sample representativeness. This equalises different participation rates in the federal states and school types so that the distribution corresponds to the population. Due to the weighting, all three age categories and the binary gender categories of girls and boys are included in the analyses in equal parts from the 2017/18 survey cycle onwards. In the 2022 HBSC survey cycle, gender was not recorded exclusively in binary form for the first time, with 1.7 % of respondents indicating the gender category gender diverse. This was taken into account in the weighting of the 2022 data, while girls and boys were weighted equally (49.2 % each; participants who did not specify their gender were excluded). Further details on the weighting of the data can be found in the article by Winter & Moor et al. [20].

A Mann-Whitney U test was used to compare the HL between the 2017/18 and 2022 survey cycles. The effect size was assessed using the Pearson correlation coefficient r, which can be interpreted as low (*r* 0.1 – < 0.3), medium (*r* 0.3 – < 0.5), or strong (*r* ≥ 0.5) according to common conventions [27]. It should be noted that due to the limited number of available time points, it is not yet possible to depict a trend, only initial tendencies. The bivariate and multivariate analyses are based on the current data set for the year 2022, focusing on a) the relationship between HL and sociodemographic and socioeconomic characteristics and b) the relationship between HL and psychosomatic complaints among students. Cross-tabular analyses with chi-square tests were used to analyse bivariate correlations. In cases with cell populations below n = 5, Fisher’s exact test (FET) was used. The multivariate evaluation was performed using binary-logistic regression analyses. Statistical adjustments were made to the calculated correlations for the characteristics of age, gender, history of migration, school type, and family affluence. The results are reported as odds ratios (OR) and 95 % confidence intervals (95 % CI). All analyses were based on a significance level of p < 0.05.

## 3. Results

### 3.1 Health literacy: a comparison between 2017/18 and 2022

**Figure 1:**
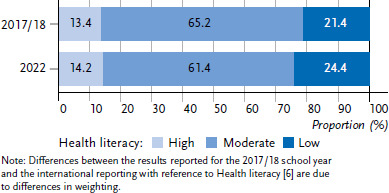
Health literacy of students in a comparison of the HBSC survey cycles 2017/18 (N = 3,707, n = 1,856 female and n = 1,851 male) and 2022 (N = 4,839, n = 2,400 female, n = 2,359 male, and n = 80 gender diverse) Source: HBSC Germany 2017/18, 2022

In the 2022 survey, 24.4 % of students were found to have low HL, 61.4 % moderate HL and 14.2 % high HL. For the 2017/18 school year, a comparison shows that low HL is indicated for fewer students (21.4 %), whereas for a larger proportion the HL can be described as moderate (65.2 %) (Figure 1). The difference between the survey cycles is statistically significant (p < 0.05), although the effect size is small (*r* < 0.1).

### 3.2 Sociodemographic and socioeconomic differences in health literacy

With regard to gender-specific differences in HL, the results show that students who have indicated that they belong to the ‘gender diverse’ category are the most likely to be assigned low HL (51.2 %). Female (22.6 %) and male (25.2 %) respondents, on the other hand, hardly differ from each other. There are also age-specific differences in HL at the expense of younger respondents. 11-year-olds are the most likely to have low HL at 27.0 % (13-year-olds: 24.9 %, 15-year-olds: 21.8 %). However, at 19.1 %, the proportion of those with high HL is also highest in the youngest age group. With regard to migration status, the results show that students with a one-sided (27.3 %) and two-sided migration history (27.1 %) are more likely to have limited HL than students without a history of migration (22.8 %).

**Figure 2:**
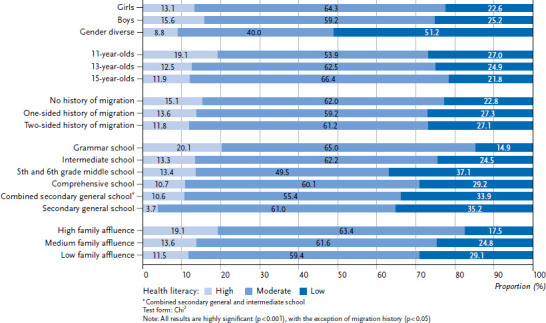
Health literacy of students, differentiated by gender (n = 4,839), age (n = 4,839), type of school (n = 4,840), migration background (n = 4,698), and family affluence (n = 4,763), 2022 Source: HBSC Germany 2022

In terms of socioeconomic differences, the analysis shows a clear educational gradient. Students at 5th and 6th grade middle schools (37.1 %), secondary general schools (35.2 %) and combined secondary general and intermediate schools (33.9 %) are the most likely to have limited HL. In contrast, grammar school students are the least likely to have low HL in a comparison of school types (14.9 %). Differentiation according to family affluence clearly shows that students with a low level of affluence (29.1 %) have more difficulties in dealing with health information than students in the medium (24.8 %) and high affluence categories (17.5 %). Figure 2 illustrates the results of the bivariate analyses.

The results of the binary-logistic regression analysis largely support the findings of the cross tabulation (Table 1). The gender-specific analysis shows that adolescents in the ‘gender diverse’ category are at a 4.12 times higher risk of low HL than female respondents. Girls and boys do not differ from each other. Furthermore, 11-year-olds show lower HL than 15-year-olds, whereas no differences are evident for 13-year-olds compared to 15-year-olds. The binary-logistic regression shows no differences for presence or absence of migration history. In the comparison of school types, a higher risk of lower HL can be derived for students from school types other than grammar school (secondary general school, 5th and 6th grade middle school, combined secondary general and intermediate school) (OR = 2.80 to OR = 2.92). With regard to family affluence, students from low and medium affluence backgrounds are at a higher risk of having a low HL than respondents with a high level of affluence.

### 3.3 Associations with psychosomatic complaints

In the current survey, 40.8 % of students stated that they were affected by at least two of the recorded complaints at least once a week.

**Figure 3:**
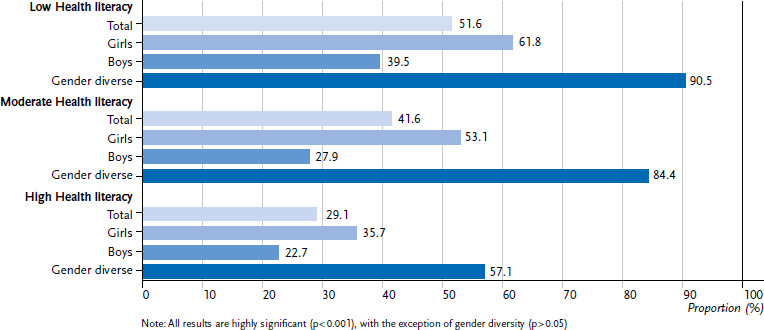
Proportion of students with a high level of psychosomatic complaints (at least two complaints at least weekly), differentiated by level of health literacy, 2022 (N = 4,835, n = 2,399 female, n = 2,355 male, and n = 81 gender diverse) Source: HBSC Germany 2022

A differentiated analysis of psychosomatic complaints shows that students with low HL (51.6 %) are more frequently affected by a high psychosomatic burden than students with high HL (29.1 %). Furthermore, when stratified by gender, it can be seen that female and gender diverse respondents, especially those with low HL, are more likely to have a high level of psychosomatic complaints. It should be noted that the results of the bivariate analysis for gender diverse respondents are not statistically significant (Figure 3). This result is confirmed by the adjusted analyses of the binary-logistic regression analysis (Table 2). After controlling for the sociodemographic and socioeconomic characteristics, it emerges that students with low HL have an increased risk of a high psychosomatic burden by a factor of 2.64. The gender-differentiated analyses of the psychosomatic complaints show a risk factor increase to 2.68 and 9.80 for girls and gender diverse respondents, respectively.

## 4. Discussion

###  

#### Summary

In comparison to the survey from the 2017/18 school year, an increase of around three percent in the proportion of students with low HL was shown for the survey year 2022. For the current HBSC survey, differences in HL can be identified according to gender (at the expense of gender diverse children and adolescents), age (at the expense of younger students), school type (at the expense of students who do not attend grammar school), and family affluence (at the expense of students with a low level of affluence). Even after correcting for the above-mentioned social background characteristics, low HL is associated with a higher burden of psychosomatic complaints.

#### Strengths and limitations

The HBSC study comprises a large representative sample of children and adolescents aged 11, 13, and 15. It is the only national study that focusses on health in the school context and is internationally comparable. HBSC uses validated instruments to assess the health and HL of children and adolescents. In addition, for the first time in a national context, the relationship between health and psychosomatic complaints among students was analysed.

One limitation is that it is not yet possible to analyse trends in the data on HL, as only two survey dates are currently available. If HL is recorded in future survey cycles, initial trend analyses can be carried out. It should be noted that no causal statements can be made due to the cross-sectional study design.

Overall, a cautious interpretation of the HL results is required, as the HLSAC scale is a self-assessment tool for children and adolescents and is therefore subject to certain limitations (e.g. tendency to over/underestimate own abilities) [6]. When presenting results, it should always be borne in mind that the levels are based on subjective assessments of students’ confidence in their ability to deal with health information. The applicability of the scale with regard to younger students is also critically discussed in the literature. Due to the complexity of the HL construct and the items used here, it can be assumed that 11-year-old students have greater difficulties in answering the questions, which is associated with a higher proportion of missing values [11, 20].

The FAS was used to describe socioeconomic differences in HL. The FAS is a regularly updated and validated instrument that is generally suitable for mapping the socioeconomic situation of children and adolescents. However, as described by Moor et al. [28] in this issue of the Journal of Health Monitoring, when interpreting the results, it must be considered that the indicators used do not necessarily provide a picture of economic prosperity in view of changing living conditions and norms (e.g. a family may deliberately choose not to own a car in the context of the climate crisis). The use of family affluence as an indicator of social status has been criticised in other studies. It is known that family affluence becomes a less significant indicator when youth-specific indicators (such as school type as indicator of the adolescent’s educational level) are added. There is also debate about the extent to which the material wealth measured by the FAS adequately reflects the actual socioeconomic situation of the family or the adolescent, particularly in western industrialised nations [29].

#### Interpretation

The difference in the frequency of low HL between the survey cycles was comparatively small at three percentage points. The tendency towards an increase in the proportion of low HL among students in 2022 should be discussed in the context of the COVID-19 pandemic. The increase in difficulties in dealing with health information appears plausible against the background of the infodemic caused by the COVID-19 pandemic. This refers to the sharp rise in information of different qualities. This increasing amount of heterogeneous information has made it more and more difficult for children and young people to navigate and orientate themselves in a highly dynamic information space [30]. The findings of the HBSC study are in contrast to the results of a population-representative study, which indicates an increase in sufficient HL during the course of the pandemic [10]. However, the study points to strong differences between individual population groups, although only the adult population was represented in the study [31]. In the course of the pandemic, the importance of health from a social perspective increased significantly. It is possible that children and adolescents reached the limits of their HL in the context of the pandemic, perhaps in part because parents played a major role in protecting against infection and adolescents had less impact on decision-making [32]. This is also shown by the results of the HLS-COVID-19 study, which indicates uncertainty due to too much information about the COVID-19 pandemic, especially for young adults [33]. However, as described above, the results should be interpreted with caution in light of the small changes in HL.

In contrast to the previous results of the HBSC study, the present analyses did not reveal any differences in HL between female and male respondents [6, 11]. Other studies for the target group of children and adolescents likewise did not find any gender-specific differences in HL (e.g. [2]). It should be noted that the majority of previous studies refer to the binary understanding of gender (girls/boys). This article shows that gender diverse respondents belong to a vulnerable group with lower HL than for girls and boys. The number of studies on HL in gender diverse people in Germany is still very limited [34]. Initial studies indicate that transgender and non-binary people often report difficulties in dealing with health information [35]. One possible explanation is that people with greater or more specific demands on the healthcare system have a high need for information. They are therefore more challenged with regard to their HL, which is why difficulties in dealing with health information are more readily apparent [36]. In principle, the available results for the group of gender diverse children and adolescents should be interpreted with caution in view of the small number of cases (1.7 %; n = 80).

The results on the age-specific consideration of HL are consistent with previous results of the HBSC study. Younger students tend to have greater limitations in HL [6, 11]. This seems plausible considering developmental differences between the age groups. Interestingly, the results also show that 11-year-olds are the age group that most frequently rates their HL as ‘high’. It is possible that younger children and adolescents tend to overestimate their own abilities, as many decisions were taken by caregivers (e.g. parents) or organisations (e.g. schools) during the pandemic while children and adolescents had to support these without being able to make their own decisions. It can also be assumed that 13- and 15-year-old students assess their own competences more realistically than younger students for developmental reasons. Further research is needed to provide a more detailed assessment. It also bears repeating that the use of the survey instrument for 11-year-olds can lead to misjudgements due to comprehension difficulties [11]. Here too, further research is needed to confirm the assumptions described.

Differentiated by migration history, the results of the multivariate analysis show no differences in HL. This seems to contradict other studies [12, 37]. Language barriers of people with a migration history are often cited as a reason for limited HL [38]. However, it should be noted that studies that specifically investigate the HL of people with a history of migration do not find that this population group is more vulnerable with regard to HL [39]. In addition to the country of origin, differences should therefore be discussed with regard to the living environment, biography, and social structure in connection with HL [38]. This ties in with current calls for the further development of public health research on people with a history of migration, which advocate taking into account underlying individual variables in the evaluation (e.g. country of birth, length of stay, nationality(ies), residence status, language skills) instead of a dichotomous evaluation of the migration history [40].

Stratification by type of school shows, in line with other studies, that students from types of school other than grammar school are at a higher risk of low HL [11, 41]. The determinants of HL discussed in previous research include functional competences, such as literary and numerical skills, which are potentially more pronounced in grammar school students than in students from other types of school (see e.g. [12, 13]). This contrasts with the results of Seifert et al. [42], who found no significant correlation between cognitive abilities and reading comprehension or subjectively measured HL. On the other hand, objective measures of HL showed medium positive correlations with these abilities [42]. It should be noted that, in addition to cognitive abilities, other characteristics should also be considered as determinants of HL, which potentially differ between students of different school types (e.g. self-efficacy) [43].

The consideration of stratification according to family affluence is consistent with the results of other studies, according to which the economic situation is a significant explanatory characteristic for the manifestation of health inequality, in line with current theories on health inequality [6, 12, 44, 45]. The results emphasise the need for target group-specific measures to strengthen HL, which relate to the needs of children and adolescents from different socioeconomic groups.

The relationship between HL and psychosomatic complaints was analysed for the first time in a national context in this article. It was found that students for whom the analyses revealed low HL were more frequently affected by psychosomatic complaints than those for whom high HL was found. This is consistent with the results of Zhang et al. [14]. When stratified by gender, it was also found that female and gender diverse respondents, especially those with low HL, were more likely to have a high level of psychosomatic complaints. However, due to the number of cases, the effect for gender diverse respondents was not statistically significant. Further details on the classification of gender-specific differences in psychosomatic health can be found in the article by Reiß & Behn et al. [19]. The findings of this study are thus in line with the results of many other studies that illustrate the relationship between HL and indicators of health in children and adolescents (e.g. [6, 46, 47]). As Okan et al. [13] illustrate, the relationship between HL and (psychosomatic) health is mediated indirectly via health-related attitudes and behaviours. These are considered important determinants of mental and psychosomatic health. It should always be borne in mind that both HL and health-related indicators are subject to a social gradient. Students with a low socioeconomic status are therefore exposed to multiple disadvantages and are at a higher risk of having their HL and psychosomatic health impaired [13, 47].

#### Conclusions

The results of the HBSC study emphasise the need for measures to strengthen children’s and adolescents’ HL. To this end, measures to promote HL should be offered in all settings relevant to young people (including day-care centres, children’s and youth centres, sports clubs, schools, etc.). Corresponding strategies should not only focus on the individual abilities and resources of adolescents, but also on the (organisational) conditions under which HL can develop [48, 49]. A suitable setting for this is the school, where extensive health promotion and prevention activities have already been carried out in recent years [50]. In this context, HL should by no means be introduced as a new or independent intervention strategy, but should be integrated into existing holistic approaches to health promotion and good healthy schools [51]. Some German federal states have comprehensive structures and programmes in place. Holistic approaches to school health promotion in particular focus on the level of individual competences and on the level of school structures and processes, thus providing a meaningful basis for the integration of new approaches. Initial considerations for combining HL with the overarching approach of health promotion [13] and for linking it with existing curricula [52] have already been made. In addition, offers and tools are available that can be used to promote HL of students and the organisation [53, 54].

As found in the present study, students show differences in HL depending on their social status and the type of school they attend. Accordingly, intervention activities in a school setting should be differentiated according to need. In order to counteract the so-called ‘prevention dilemma’, schools with a particularly high need for measures to strengthen health and HL should be prioritised (especially secondary general schools, intermediate schools, comprehensive schools and 5th and 6th grade middle school). The provision of health information for children and adolescents in simple language is one way of creating target group-appropriate programmes [55]. Due to pandemic-related challenges, school health promotion programmes could not be implemented or maintained as planned in recent years [56]. With this in mind, efforts should be made to consolidate existing services, even in exceptional situations such as the COVID-19 pandemic, so that health-competent schools can be created at an organisational level [49]. Schools should be supported in this task by a network of other actors in prevention and health promotion [57].

## Key statement

- Compared to the 2017/18 survey (21.4 %), slightly more students have low health literacy in 2022 (24.4 %).
- Younger students have greater difficulties in dealing with health information, while girls and boys do not differ from each other.
- There is a social gradient in health literacy at the expense of students from schools other than grammar schools and those from socioeconomically disadvantaged families.
- Low health literacy is associated with a high level of psychosomatic complaints among the students surveyed.
- There is a need for target group-specific offers to strengthen young people’s health literacy, focusing on individual and organisational aspects.

## Figures and Tables

**Table 1:** Multivariate logistic regression on the probability of occurrence of low health literacy among students (N = 4,593, n = 2,371 female, n = 2,145 male, and n = 77 gender diverse) 2022 Source: HBSC Germany 2022

	Low health literacy		
	OR	(95 % CI)	p-value
**Gender**			
Girls (Ref.)	–	–	–
Boys	1.10	(0.95 – 1.27)	0.169
Gender diverse	**4.12**	**(2.58** – **6.59)**	**< 0.001**
**Age group**			
15-year-olds (Ref.)	–	–	–
13-year-olds	1.16	(0.98 – 1.37)	0.084
11-year-olds	**1.34**	**(1.12 – 1.60)**	**0.001**
**Migration history**			
None (Ref.)	–	–	–
One-sided	1.21	(0.98 – 1.50)	0.077
Two-sided	1.01	(0.85 – 1.19)	0.945
**Type of school**			
Grammar school (Ref.)	–	–	–
5th and 6th grade middle school	**2.80**	**(1.77** – **4.45)**	**< 0.001**
Secondary general school	**2.92**	**(2.26** – **3.78)**	**< 0.001**
Intermediate school	**1.88**	**(1.52** – **2.33)**	**< 0.001**
Comprehensive school	**2.35**	**(1.92** – **2.88)**	**< 0.001**
Combined secondary general and intermediate school	**2.87**	**(2.34** – **3.52)**	**< 0.001**
**Family affluence**			
High (Ref.)	–	–	
Medium	**1.28**	**(1.04 – 1.57)**	**< 0.05**
Low	**1.37**	**(1.07 – 1.75)**	**< 0.05**

OR = Odds ratio, CI = confidence interval, Ref. = reference group

Bold: Significant result (p < 0.05)

**Table 2:** Multivariate logistic regression for the prediction of a high level of psychosomatic complaints among students, differentiated by health literacy and gender, 2022 (N = 4,586, n = 2,371 female, n = 2,138 male, and n = 77 gender diverse) Source: HBSC Germany 2022

	High level of psychosomatic complaints		
	OR	(95 % CI)	p-value
**Health literacy**			
High (Ref.)	–	–	–
Moderate	**1.61**	**(1.32** – **1.95)**	**< 0.001**
Low	**2.64**	**(2.12** – **3.29)**	**< 0.001**
**Gender**			
Boys (Ref.)	–	–	
Girls	**2.68**	**(2.36 – 3.04)**	**< 0.001**
Gender diverse	**9.80**	**(5.19 – 18.51)**	**< 0.001**

Bold print: Significant result (p < 0.05)

OR = Odds ratio, CI = confidence Interval, Ref. = reference Group

Results adjusted for the variables gender (only for health literacy), age group, migration history, type of school, family affluence
